# Bedside ultrasound as a screening test for the diagnosis of catheter-related bloodstream infection (CRBI)

**DOI:** 10.1007/s40477-020-00538-8

**Published:** 2021-02-09

**Authors:** Chiara de Sio, Mario Venafro, Giampiero Foccillo, Riccardo Nevola, Lucio Monaco

**Affiliations:** 1 Internal Medicine, University of Campania Luigi Vanvitelli, Naples, Italy; 2 Emergency Medicine, CTO Hospital, Naples, Italy; 3 Emergency Medicine, San Paolo Hospital, Naples, Italy

**Keywords:** Catheter-related blood stream infections, Vascular devices, Vascular ultrasound, ROC curve

## Abstract

**Purpose:**

Between 15 and 30% of all nosocomial bacteremias and sepsis are associated with the use of intravascular devices. Catheter-related bloodstream infections (CRBI) are infections in which the organism identified in the blood is also present on the tip of the catheter itself or in a blood sample taken through it. The aim of the study was to evaluate the role of ultrasound in the diagnosis of infections related to the use of central catheters.

**Methods:**

Between January 2018 and June 2019, we carried out a prospective study on 36 patients with a central catheter, such as a central venous catheter (CVC), a central catheter with peripheral insertion (PICC), or a fully implanted central venous catheter (PORT-a-cath) and who had signs and symptoms of infection. These patients were submitted to an ultrasound of the catheter upon arrival in the ward in case of suspected infection, or at the time of the onset of signs and symptoms of infection (if these arose during hospitalization). Patients with a central catheter but without signs and symptoms of infection were not included in the study.

The end point of the study was to evaluate sensitivity (SENS), specificity (SPEC), positive and negative predictive value (PPV-NPV) and overall diagnostic accuracy (ODA) of ultrasound in the diagnosis of CRBI through Receiver Operating Characteristic (ROC) curve analysis.

**Results:**

US showed a SENS of 94%, a SPEC of 84%, a PPV of 84%, an NPV of 94% and an ODA of 88.8% for the diagnosis of CRBI.

**Conclusions:**

Preliminary data from our study show that US of intravascular devices has a high SENS and SPEC in the diagnosis of CRBI, and can, therefore, be used as a valid tool to decide whether to remove the device early or leave it in place.

## Introduction

Vascular access is invaluable in the treatment of hospitalized patients. It provides a durable and long term solution while saving patients from repeated needle sticks for peripheral IVs and blood draws [1]. It is an important tool in the treatment of critically ill patients. In the USA, over 15 million catheter/days/year are recorded in intensive care units (ICUs) alone (i.e. the total number of days of exposure to CVCs among all patients in the selected population during the selected time period) [2]. Placement of a CVC is a basic skill for every medical specialty; however it is not without risks. Complication rates during CVC placement are reported as high as 15–33% [3, 4]. Even after successful placement, having an indwelling CVC put the patients at risk of catheter-related bloodstream infections (CRBI).

Each year in the USA, CVCs cause an estimated 80,000 CRBI in ICUs [5]. A total of 250,000 cases of CRBI have been estimated to occur annually, if entire hospitals are assessed, resulting in up to 62,000 deaths among patients in hospitals according to a recent meta-analysis [6].

High rates of CRBI (26 times higher than that of general populations) have also been described in patients undergoing dialysis, particularly in patients with CVCs (20-fold higher risk) rather than arteriovenous fistula [7].

About 7.5% of health care endocarditis cases were associated with indwelling catheter infection [8], and 15–30% of all nosocomial bacteremias are associated with intravascular devices (both central and peripheral venous catheters) [9]. CRBI have also been observed in patients with a central catheter with peripheral insertion (PICC) [10] and with a fully implanted CVC (PORT-a-cath) [11].

CRBI are defined as infections in which the organism identified in the blood is also present in significant amounts on the tip of the catheter itself or in a blood sample taken through it. Three approaches are currently available for the diagnosis of CRBI:

- semi-quantitative sample of the catheter tip (gold standard): the diagnosis of CRBI is confirmed if the same germ is isolated from the catheter tip and the blood sample, and if the growth on the catheter tip is > 15 colony-forming units (CFU) in 24 h [12];
- differential quantitative blood samples: the diagnosis of CRBI is confirmed if the same germ is isolated from the blood sample of the catheter and from the peripheral blood sample and if the colony count of the former is at least 3 times higher than the count of the latter [12];
- differential time in positive sample: the diagnosis of CRBI is confirmed if the same germ is isolated from the blood sample of the catheter and from the peripheral blood sample and the growth is detected at least two hours before in the blood coming from the catheter [12].

A rapid diagnosis of CRBI is very important: efforts should be made to prevent underdiagnosis of CRBI as this can lead to inappropriate therapy without catheter removal. On the other hand, efforts should be made to prevent overdiagnosis to avoid unnecessary catheter loss (it is important to have a valid vascular access in patients at risk of septic shock).

Since its introduction, ultrasound has increased the success and safety of central and peripheral venous access. Indeed, it minimizes the risk of complications such as repeated punctures, accidental punctures of arterial vessels, pneumothorax, etc.[13, 14].

Ultrasound also allows the identification of thrombus associated with catheters [15, 16]. In an infection associated with a catheter, the bacteria are deposited at the level of the tip or along the course of the catheter, forming fibrinous vegetations similar to thrombus in which germs are trapped [16]. The proliferation of these microbes can therefore transform the thrombus into an intravascular abscess; this condition is known as septic thrombophlebitis [16].

The diagnosis of septic thrombophlebitis requires evidence of thrombosis in the cannulated vessel and persistent sepsis with no other evident source of infection. Treatment includes catheter removal and 4–6 weeks of systemic antibiotic therapy. If not recognized and treated, septic catheter-associated thrombophlebitis has a high mortality risk.

## Aim

The aim of our study was to evaluate the role of ultrasound in terms of sensitivity (SENS), specificity (SPEC), positive predictive value (PPV), negative predictive value (NPV) and overall diagnostic accuracy (ODA), as a screening test in the early recognition of catheter-associated septic thrombophlebitis, to treat this condition as quickly as possible and to reduce infection-related mortality [16].

## Materials and methods

To evaluate the use of ultrasound as a screening test in the early diagnosis of CRBI, we carried out a prospective study on patients consecutively admitted to the Department of Internal Medicine of the University of Campania Luigi Vanvitelli in Naples, the Emergency Department of the Orthopedic Trauma Center (CTO) in Naples, and the Emergency Department of San Paolo Hospital in Naples, in the period January 2018 to June 2019, who had a central catheter, such as CVC, PICC or PORT-a-cath, and with signs and symptoms of infection (fever, elevated white blood cell count, elevated CRP and/or PCT, etc.) or who developed signs and symptoms of infection during hospitalization, with a suspicion of CRBI. We enrolled 36 patients. There were 16 males (median age 71.5, range 63–88 years) and 20 females (median age 59.5, range 19–88 years).

Ultrasonography was performed by one experienced operator with more than five years of practice in ultrasonography (CdS).

We used a multifrequency linear probe (7–13 MHz) and the evaluation was made at the site and along the course of the vascular device.

**Fig. 1:**
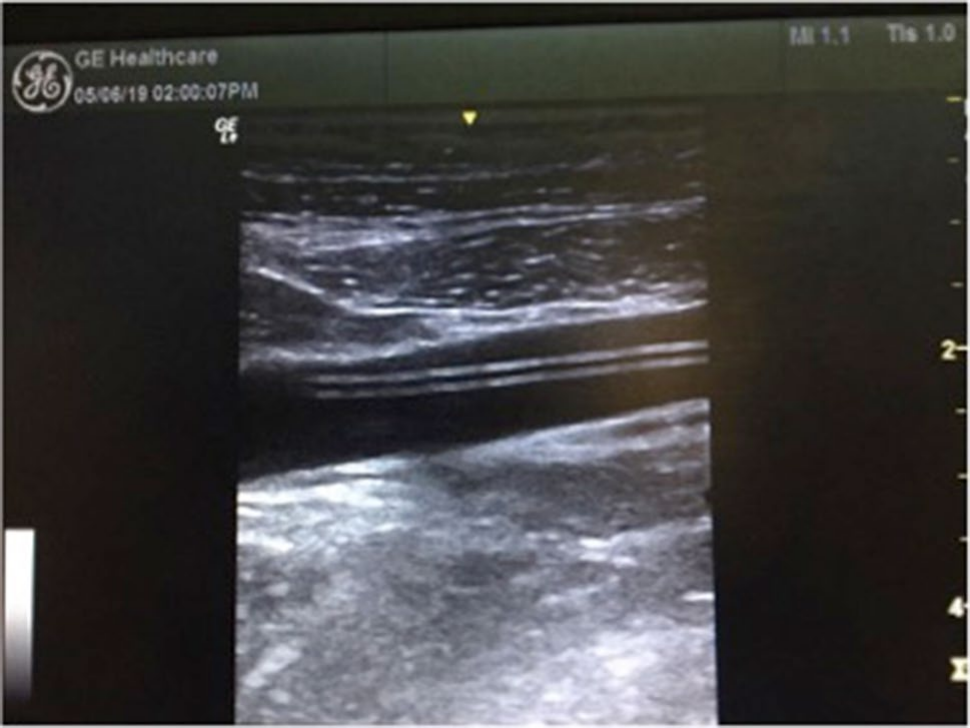
The image, obtained using a multifrequency linear probe (7–13 MHz), shows the typical aspect “binary like” of a PICC in the right brachial vein

Ultrasonography was defined as negative when the vascular device was clearly visible and showed the typical aspect “binary like” (Fig. 1); it was considered positive (with suspicion of infected catheter) when:

1. echogenic material was clearly visible in the vessel (Fig. 2)

**Fig. 2:**
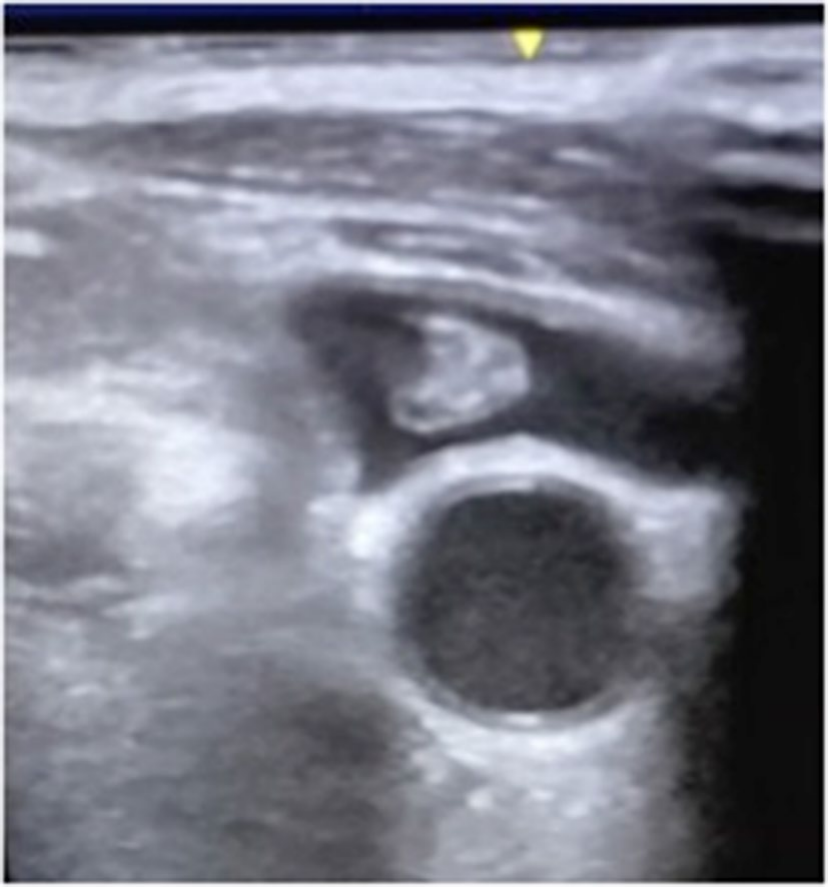
The image, obtained using a multifrequency linear probe (7–13 MHz), shows the presence of echogenic material in the left jugular vein
2. the “binary like” aspect of the vascular device was interrupted and its surface and/or lumen partially (Fig. 3a) or totally (Fig. 3b) occupied by echogenic material.

**Fig. 3:**
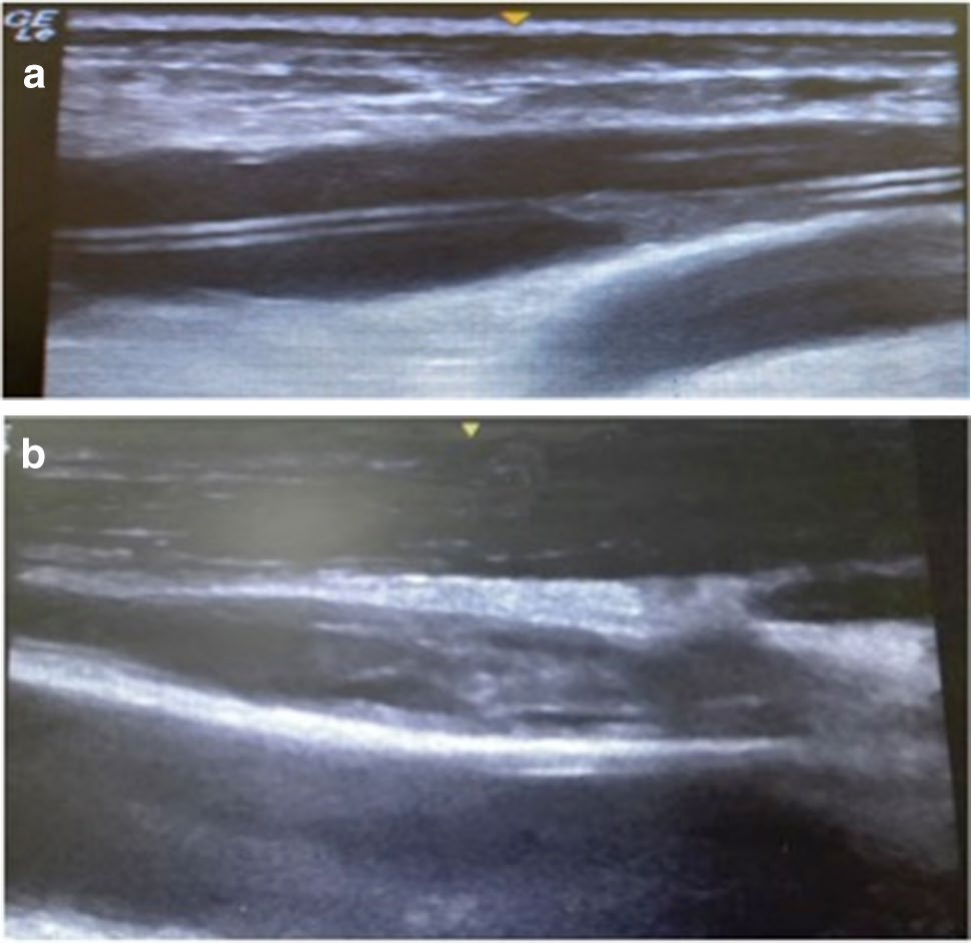
**a** The image shows the presence of echogenic material attached to the surface of the PICC (it is still possible to see the “binary like” aspect of the catheter). **b** In this image the lumen of the catheter is totally occupied by echogenic material

The ultrasound of the catheter was performed upon the patient’s arrival in the ward if there was a suspected infection, or at the time of onset of symptoms of infection (if these arose during hospitalization), at the same time of blood samples. Ultrasound of the catheter was not performed in patients without signs of infection (as the presence of such signs is a necessary inclusion criterion for the study).

From each patient was taken: blood samples by a peripheral vein, blood samples by the central catheter, and central catheter tip cultures.

In febrile patients in which ultrasonography was considered positive, the catheter was removed without any anticoagulation therapy.

The following data were included in a database: age, sex, comorbidity, temperature (T), heart rate (HR), respiratory rate (RR), mean arterial pressure (MAP), Glasgow coma score (GCS), Apache score, hematocrit (Ht), white blood cells (WBC), sodium (Na), potassium (K), creatinine, pH, PaO2, FiO2, HCO3–, type and site of vascular catheter, positivity or negativity of blood sample (both by peripheral vein and central catheter), positivity or negativity of central catheter tip cultures, type of microorganism isolated, and possible infection in other sites.

We used the results obtained from the central catheter tip cultures as the gold standard for the diagnosis of CRBI.

Statistical analysis was performed using the Mann–Whitney *U* test to evaluate differences between the two groups of patients (US positive and US negative) and receiving operator curves (ROC curves) were used to evaluate SENS, SPEC, PPV, NPV and ODA of US diagnosis of suspected infected vascular device.

The local ethics committee gave approval for this study.

## Results

Of the 36 patients enrolled, 20 had CVC (55.5%), 13 had PICC (36.1%) and 3 had PORT (3.3%).

US showed suspected infection of the vascular device in 19 of 36 patients (52.7%), while in 17 patients (47.2%) the exam was negative.

The clinical and laboratory data of patients, analyzed with the Mann–Whitney *U* test and divided according to ultrasonography positivity/negativity, were statistically homogeneous (*p* > 0.05, see Table 1).

**Table 1:** The table shows the clinical and laboratory characteristics of our study population divided into patients with US positive and negative findings

	US negative (17)	US positive (19)	*P* value
Age	70 ± 13	65 ± 17	0.4
T	37.7 ± 2.6	38.6 ± 0.7	0.3
MAP	76 ± 12	79 ± 13	0.6
HR	97 ± 23	97 ± 17	0.8
PaO_2_/FiO_2_	334 ± 115	313 ± 105	0.5
RR	20 ± 3	24 ± 6	0.1
HCO3	27 ± 4.6	24.3 ± 6.6	0.09
pH	7.46 ± 0.05	7.42 ± 0.08	0.09
Na	141 ± 9.4	138 ± 6.9	0.5
K	3.8 ± 0.5	3.6 ± 0.4	0.2
Creatinine	1.2 ± 0.6	1.25 ± 1	0.6
Ht	32 ± 5.4	31 ± 4.9	0.6
WBC	14518 ± 8339	13553 ± 7458	0.8
GCS	14.6 ±0 .6	14.6 ± 0.6	1
APACHE II score	16.7± 5.6	16.9 ± 5	0.8
CPR	12.8 ± 9.3	15.2 ± 8.6	0.4

A Mann–Whitney *U* test found no difference between the two groups (*p* > 0.05)

The time elapsed from the implantation of the catheter to the time of examination is on average 4 months in the group with positive echo vs 4.9 months in the group with negative echo (*p* 0.53). A catheter malfunction occurred in 4 cases in the positive echo group (21%) and in one case in the negative echo group (5%) (*p* 0.26).

The cultures obtained from the tip of the vascular device, considered the gold standard, documented the infection of the device in 17 of 36 patients (47.2%). We found in 11 cases a gram-positive germ, in 2 cases a gram-negative germ, in 3 cases a Candida infection, and in 1 case a mixed infection (Candida + gram-positive germ). In 7 of these patients there was also infection in other sites with a different type of isolated germ.

**Fig. 4:**
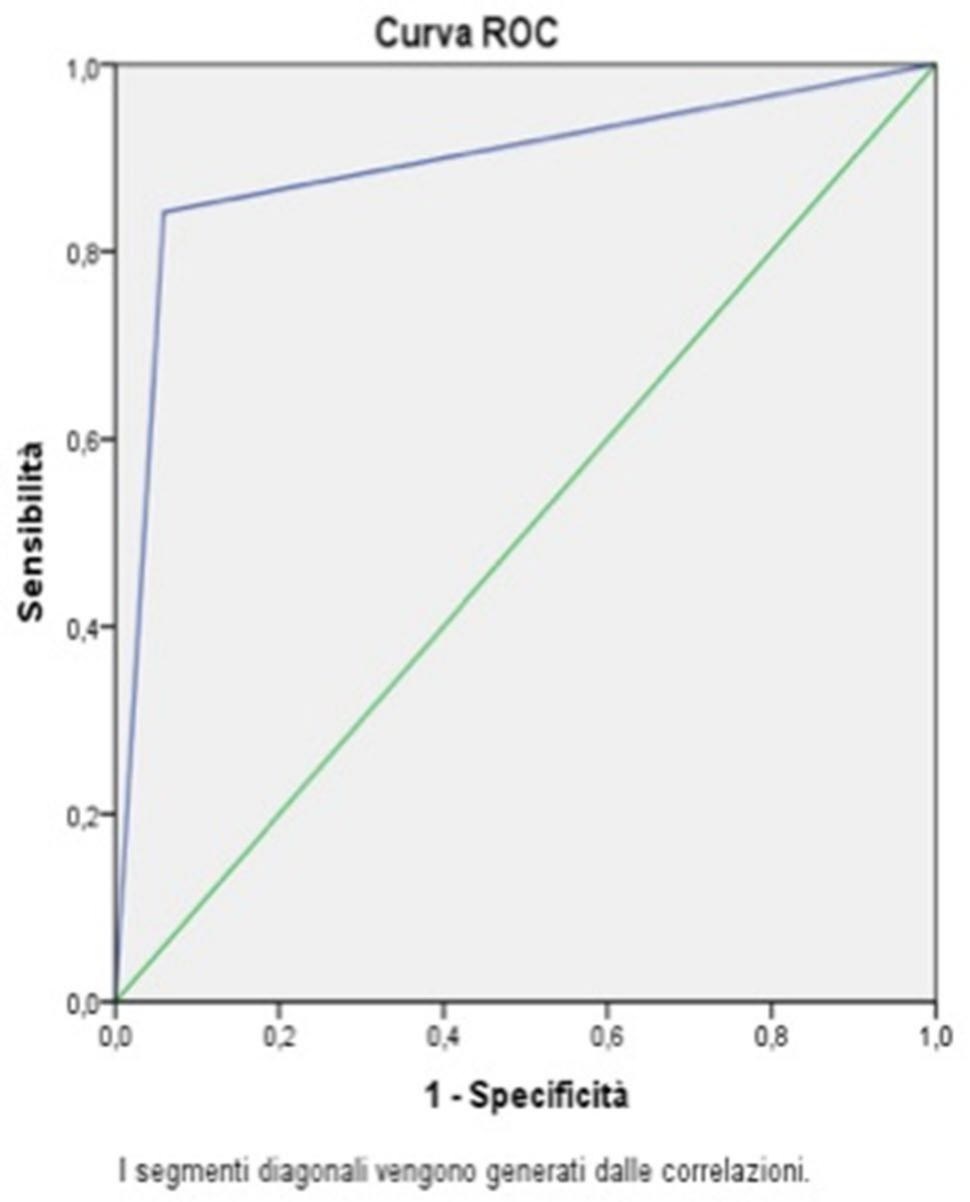
ROC curve for all types of vascular catheters

In 19 patients in which the cultures of the tip of the vascular device were sterile, the cause of infection was identified in other sites. In particular, there were 6 cases of pneumonia, 3 cases of an infected skin ulcer, 2 cases of urinary tract infection, 2 cases of biliary infection, 2 cases of post-surgery abdominal infection, 2 cases of viral infection, 1 case of endocarditis, and 1 case of soft tissue infection. US diagnosis of infected vascular device showed 3 false positives and 1 false negative result with a SENS of 94%, a SPEC of 84%, a PPV of 84%, an NPV of 94%, and an ODA of 88.8% (Fig. 4).

**Fig. 5:**
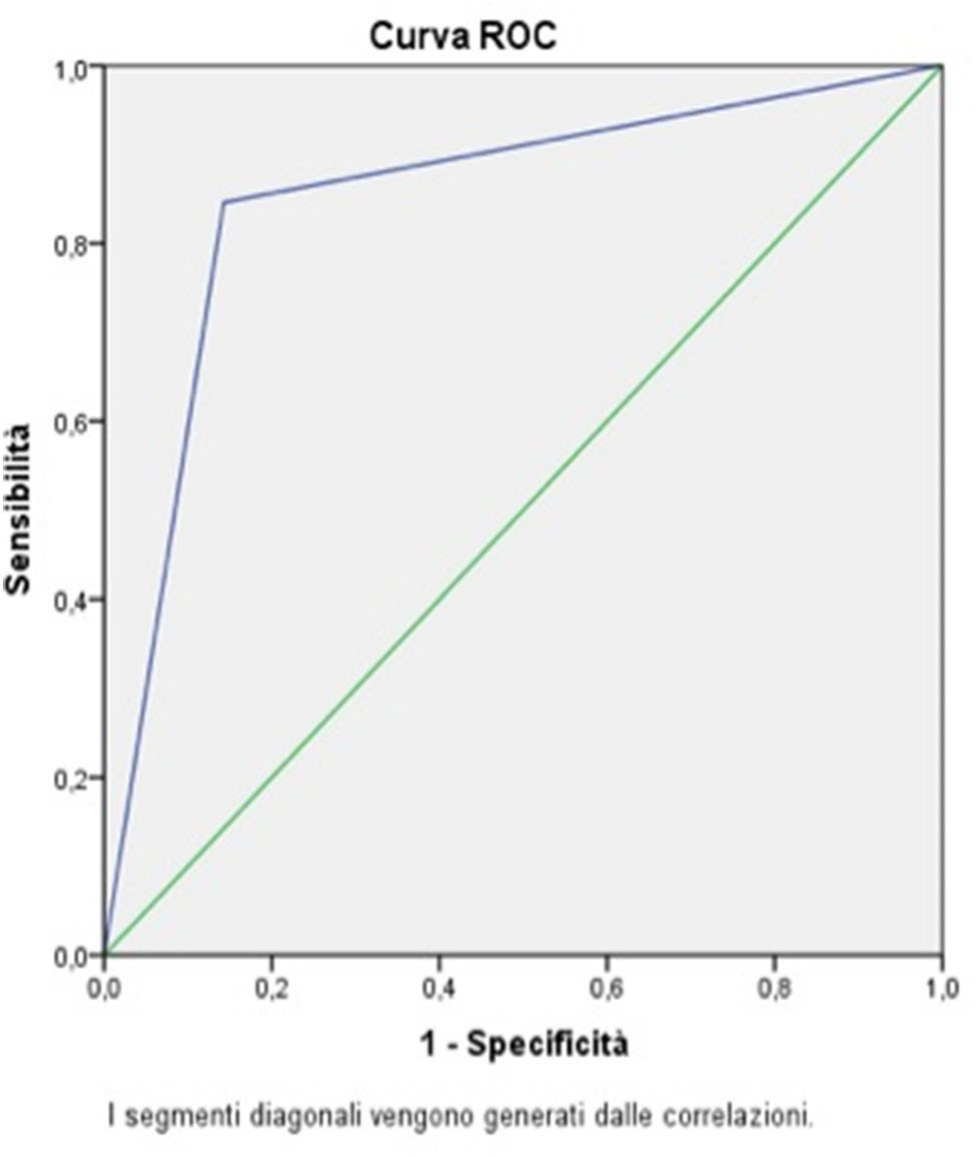
ROC curve for CVCs

In the 20 patients with CVCs, US showed 2 false positives and 1 false negative with a SENS of 91%, a SPEC of 75%, a PPV of 84%, an NPV of 85%, and an ODA of 85% (Fig. 5).

**Fig. 6:**
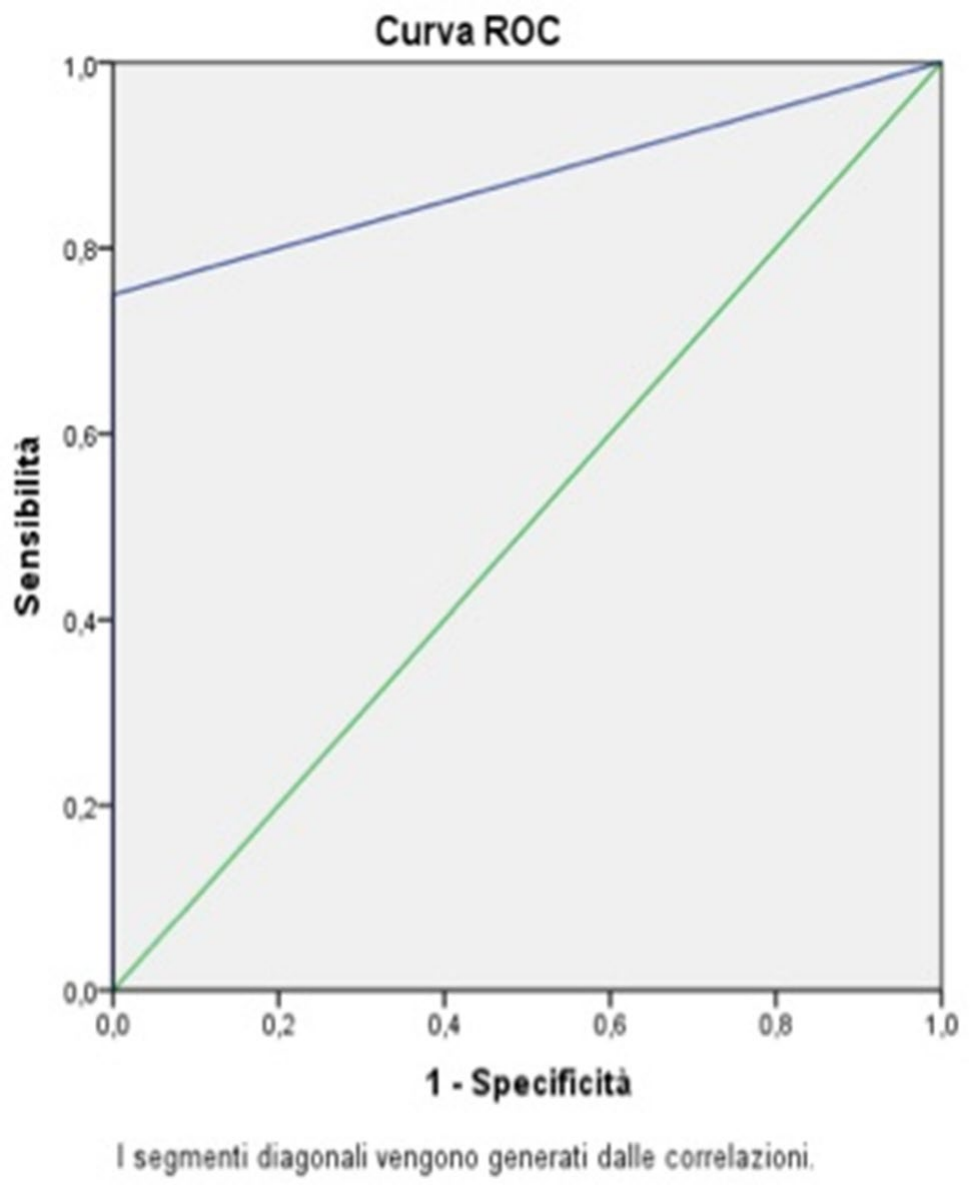
ROC curve for PICCs

In the 13 patients with PICCs, US showed 1 false positive with a SENS of 100%, a SPEC of 90%, a PPV of 75%, an NPV of 100%, and an ODA of 92% (Fig. 6).

Only a small group in our series (3 patients) had a PORTs device. In this group, US diagnosis showed no false positive and negative results, so SENS, SPEC, PPV, NPV and ODA were 100%.

## Discussion

CRBI are mostly preventable hospital-acquired conditions that contribute substantially to morbidity, mortality and health costs in all age groups and diagnoses [2]. The incidence rate of CRBI is different as studies report rates ranging from 4 to 25% of inserted catheters [3–5].

Clinical findings are unreliable for establishing the diagnosis of CRBI because of their poor SENS and SPEC; the most sensitive clinical finding, fever, has poor SPEC, while inflammation or purulence around the insertion site of a vascular device has greater SPEC but poor SENS [17, 18].

A blood workup usually includes a complete blood count, blood cultures, erythrocyte sedimentation rate and a C-reactive protein test (CRP).

Laboratory criteria for diagnosing CRBI are clear but differences in definitions and methodologies among various studies have made data difficult to compare [18, 19]; moreover a blood culture will take up to 5 days and antimicrobials will be started [6].

Recently, procalcitonin has been used as a surrogate for CRBI among hemodialysis patients [20] and pediatric patients [21]. In the study by Hamada et al. [20] in 31 hemodialysis patients, SENS and SPEC of procalcitonin levels for the diagnosis of CRBI were 94% and 100%, respectively, but one overt limitation of this study was the heterogeneity between patients regarding their original kidney disease, which may have an impact on the results.

In the study by Ozsurekci [21] on 49 pediatric patients with suspected CRBI, SENS, SPEC, PPV,

NPV and ODA were 71%, 80%, 77%, 74% and 75%, respectively [21].

Presepsin level also has been recently suggested as a new marker of CRBI in pediatric patients; in this study the optimal cut-off value for presepsin was identified by plotting ROC curves.

The calculated cut-off value for presepsin in distinguishing patients with CRBI was 990 pg/mL, with SENS and NPV of 100%. SPEC and PPV of presepsin were 93% and 92%, respectively [22].

It is relevant to outline that, in this study, some healthy children displayed high levels of presepsin.

Earlier diagnosis of CRBI may offer the ability to initiate treatment to prevent adverse outcomes; immediate recognition of signs and introduction of proper treatment can improve the prognosis of CRBI.

Vascular ultrasound is widely used to guide the insertion of a vascular device to increase the success rate of incannulation and to minimize complications.

Ultrasound of the catheter was successfully used by Esposito et al. to monitor cases of malfunction and occlusion in patients on peritoneal dialysis [23].

In our study we evaluated the role of vascular ultrasound as a screening test in the early recognition of catheter-associated septic thrombophlebitis, to treat this condition as quickly as possible and to reduce infection-related mortality.

In 2012 Picardi et al., in a prospective study conducted on 100 acute leukemia patients, had already found that an early ultrasonographic finding of septic thrombophlebitis was the main indicator of CVC removal to reduce infection-related mortality in neutropenic patients with bloodstream infection [24].

Preliminary data from our study showed that the ultrasound examination of central catheters has high SENS and SPEC in the diagnosis of catheter-related bloodstream infections, and can therefore be used as a valid tool to decide whether to remove the catheter early or leave it in place.

Particularly, the high NPV of vascular ultrasound for diagnosing CRBI suggests that, when a CVC is judged “not infected,” we can leave it in place, avoiding loss of an important vascular device that is useful in treatment of patients with possible septic shock.

Bedside ultrasound represents a rapid, easy to perform, and reproducible screening test. It does not require waiting times (as laboratory tests and other instrumental exams do), and therefore a diagnosis can be made and the catheter can be instantly removed and therapy started as soon as a suspicion of infection arises. Two of the main limitations of our study were the absence of data regarding patients with a central catheter but without signs and symptoms of infection that were not included in our study, and the heterogeneity between patients regarding their original disease, which may have an impact on the results.
